# Organic Nitrogen in Precipitation: Real Problem or Sampling Artefact?

**DOI:** 10.1100/tsw.2001.278

**Published:** 2001-11-07

**Authors:** J.N. Cape, A. Kirika, A.P. Rowland, D.R. Wilson, T.D. Jickells, S. Cornell

**Affiliations:** Centre for Ecology and Hydrology Edinburgh, Bush Estate, Penicuik, Midlothian, UK

**Keywords:** N deposition, rain chemistry, eutrophication, precipitation sampling

## Abstract

Published observations of organic nitrogen (N) compounds in precipitation go back almost a century. Several different methods have been used to measure both the total and ionic concentrations of N. There is therefore some uncertainty as to whether reported “organic N” is real, or simply the result of uncertainties in chemical analyses or inadequate sampling methods. We found that the materials from which the collector was made (polypropylene, steel, or glass) had no significant effect on the composition of dissolved organic N (DON). The use of a biocide was found to be very important during sampling and storage of samples before analysis. We set up a network of seven collectors across the U.K., from the Cairngorms to Dorset, all operating to the same protocol, and including a biocide. Samples were analysed centrally, using proven methods. Over 6 months, organic N contributed about 20% to the total N in U.K. precipitation, but with a large variation across the country. This means that current estimates of wet deposited N to the U.K., which are based only on the ammonium and nitrate concentrations, are too small. Organic N is not an artefact, but a real problem that needs to be addressed.

### INTRODUCTION

There has been concern over recent increases in the nitrogen (N) content of precipitation, and possible eutrophication of sensitive ecosystems (both marine and terrestrial), but total N deposition is usually defined in terms of inorganic N; any contribution from organic N is ignored. European protocols for controlling N emissions and deposition have also concentrated only on the inorganic components. However, recent measurements have shown that dissolved organic N (DON) in rain can stimulate estuarine bacteria and phytoplankton, and can be an important source of bioavailable N to ecosystems[1]. DON in rain has been reported to contribute up to 70% of total N[2]. These are not new observations—organic N was reported as contributing 25% of total N in rain at Rothamsted early this century[3]. However, the amounts and proportions of organic N in precipitation vary widely. In some studies, the amount of organic N in rain is below the analytical detection limit, whereas in others it comprises more than half the total N. Data from the 1970s and 1980s summarised by Timperley et al.[4] show organic N accounting for up to 90% of total wet deposited N.

It is not easy to compare results from different studies. Some studies have used event sampling, with wet-only collectors, while others have taken weekly samples over several years. There has been no consistency in sampling methods, storage conditions, or chemical analysis. The sampling device may cause bias; Scudlark et al.[5] reported significantly lower concentrations of organic N from polyethylene than from stainless steel buckets, which they attributed to adsorption of organic N by the bucket or bacterial uptake on the container walls. However, it is significant that they did not filter their samples before analysis, so that the organic N "adsorbed" may have been in particulate form. In general, there has been no consistency in the literature over filtration of samples, so it is likely that many of the values reported as "DON" include a contribution from particulate organic N.

After collection, there may be problems with sample stability. Scudlark et al.[5] noted rapid loss of organic N from some rain samples over a period of hours, attributed to volatilisation, as there was no increase in inorganic N in the samples. Several agents have been used to preserve the integrity of samples, such as $HgCl_2$, chloroform, acid, or thymol, or samples may have been frozen for long-term storage prior to analysis. Apart from the initial losses noted by Scudlark et al.[5], loss of organic N from collected samples appears not to be a major problem compared to measured changes in $NH_4^+$ or $NO_3^-$ concentrations[6], although in this example, and in most other studies, samples may have been collected several hours, if not days, after rainfall. In samples that have not been filtered, there is the possibility of slow dissolution of particulate organic N. Freezing, or preservation with $HgCl_2$, can maintain organic N concentrations for at least a year in the laboratory[2]. There is also the possibility that inorganic N can be converted to organic N, either as particulate (growth of micro-organisms) or dissolved, during sampling and/or storage—inorganic N concentrations change with storage if not preserved[7]. There is a need for a method of preservation for both inorganic and organic N which applies during sample collection, as well as during subsequent transport and storage prior to chemical analysis.

Organic N concentrations in water samples are estimated as the difference in measured concentrations of $NH_4^+$, $NO_3^-$, and $NO_2^-$ (dissolved inorganic nitrogen = DIN), and the measured total N in the sample. Methods for analysing both DIN and total N vary widely. Ammonium ion concentrations have been measured by ion chromatography or by a range of colorimetric methods based on the indophenol blue reaction. Ion chromatography is the best method in this context, as it avoids any possible contribution of organic N in the indophenol blue method. Similarly, ion chromatography for nitrite and nitrate avoids potential interference by organic N in the colorimetric methods used. The potential for positive interference by organic N in the analysis of DIN would mean that published measurements for organic N (total N – DIN) might be underestimates.

Four methods have been used for the analysis of total N in rainwater samples: Kjeldahl N (reaction to $NH_3$ followed by titration or colorimetric analysis), persulphate digestion (usually followed by colorimetric analysis), UV oxidation, and high temperature (>1000°C) combustion with chemiluminescent analysis of NO (pyro-chemiluminescence). Some authors have directly compared analytical methods. For example, Cornell and Jickells[8] compared persulphate digestion with UV oxidation for a range of rain samples and synthetic solutions and found that results using the persulphate method were about 30% lower than for the UV oxidation method. Conversely, Scudlark et al.[5] found that persulphate oxidation gave larger total N concentrations in rain samples, and higher recoveries of pure compounds spiked into a rainwater sample, than UV oxidation.

Although doubt exists over the reliability of historical measurements of total N in precipitation, either through uncertainty over sampling methods (i.e., the possibility of microbial transformations from inorganic to organic N during sampling or storage) or the reliability of the analytical methods employed, there are well-characterised recent data which suggest that the occurrence of DON in precipitation is real, and contributes around one-third of the total measured N[2,5]. This paper describes our experimental approach to testing possible artefacts in the collection, storage, and analysis of rain samples, with the aim of establishing a robust protocol for measuring DON in precipitation.

### EXPERIMENTAL METHODS

#### Chemical Analysis

Analytical methods were tested using a range of synthetic organic and inorganic compounds thought to be characteristic of the composition of precipitation, or "model" compounds that we have used previously to test the efficiency of oxidation procedures in natural waters, namely nitrate, ammonium, urea[11], glycine, methionine[12], and 5-iminodiacetic acid. Recoveries of these compounds at concentrations typical of those found in rain or dew (range 5 to 20 μM) were tested in the presence of alternative chemical biocides chloroform or thymol (2-isopropyl-5-methyl phenol), and after storage at different temperatures.

#### *Inorganic Components*

Nitrate ($NO_3^-$) was determined by chemically suppressed ion chromatography (carbonate/bicarbonate eluant and AG4A/AS4A columns) using a Dionex DX100 system configured with a 50-μl sample loop and an autosampler. An automated continuous flow colorimetry method used for the analysis of soil extracts and persulphate digestions was also evaluated, in which nitrate is initially reduced to nitrite with Cu/hydrazine and reacted using Greiss-Ilosvay diazotization to form a coloured complex.

Ammonium ($NH_4^+$) was determined by chemically suppressed ion chromatography (sulphuric acid eluant and CG12A, CS12A columns) using a Dionex DX100 system configured with a 50-μl sample loop and an autosampler. An automated continuous flow colorimetry method used for the analysis of aqueous samples from a wide range of sources, based on the indophenol blue reaction, was also evaluated.

#### *Total Dissolved Nitrogen*

The Kjeldahl method used in early studies is based on the digestion of aqueous samples in sulphuric acid, with the addition of a salt and catalyst, followed by neutralisation, distillation, and titration. Our tests indicated that the Kjeldahl method was not suitable for the accurate determination of N in precipitation, mainly due to the high reagent/acid concentrations, which led to high and variable blanks when working with dilute solutions such as rainwater.

Alkaline persulphate oxidation in an autoclave (or sometimes a microwave oven) has superseded the Kjeldahl digestion method, and was used here followed by colorimetric determination of nitrate. We added 0.35 g potassium persulphate, 0.67 ml 0.02 M NaOH, and 3.33 ml water to a 40-ml aliquot of sample, and autoclaved at 100 k Pa and 120°C for 45 min. We also evaluated a batch UV digestion method[8] which uses a Metrohm UV digester followed by quantification of ammonium, nitrite, and nitrate products by colorimetry or ion chromatography. A few samples were analysed using a N specific HPLC detector (ANTEK 8060) in which 20-µl samples are introduced into an eluant stream, combusted in oxygen at 1050ºC, and the resultant NO detected by its chemiluminescent reaction with ozone.

### Choice of Biocide

A biocide is necessary to prevent biological deterioration of rainwater samples in the field (during collection—particularly important for weekly network samples) and while being transported and stored before analysis. N-containing compounds are particularly susceptible to biological degradation. Thymol (2-isopropyl-5-methyl phenol) was chosen as the biocide, based on earlier studies[7], because it is (relative to chloroform) nonvolatile, effective at low concentrations in solution, and presents few toxicity problems for eventual disposal (cf. Hg salts). If no biocide is applied in the field, alternative preservation techniques may be applied once the sample has been returned to the laboratory, such as the use of chemical biocides (e.g., chloroform), storage at low temperatures, or freezing.

#### *Establishing Effective Biocide Concentrations During Sampling*

A sample of rainwater was collected, filtered (ashed glass fibre), and divided into 21 aliquots. To these solutions were added seven different concentrations of thymol up to 1 g $l^{-1}$, giving three replicates per treatment. The samples were then stored covered (to exclude dust, but not sealed) in a temperate greenhouse (ca. 25°C) for 1 week to simulate storage in a rain collector in the field, before analysis for $NO_3^-$ and $NH_4^+$ (ion chromatography) and total N (persulphate digestion followed by colorimetry of $NO_3^-$).

#### *Test of Long-Term Preservation Methods*

After sample collection, time may elapse during transport and storage at the laboratory before analysis. Two studies of short-term stability (up to 3 months) were made using filtered rain samples, and one long-term (9 month) study.

#### *Long-Term (9-Month) Storage of Filtered Sample*

A sample of rainwater was modified by the addition of urea to provide ca. 70 µM organic N, filtered, and aliquots were stored in the dark at 4ºC or in a freezer at -12ºC for up to 9 months, with periodic analysis for inorganic N by ion chromatography, and for total N by UV digestion.

#### *Short-Term (3-Month) Storage of Filtered Samples*

In two separate experiments, freshly collected rain samples were filtered and stored using a variety of methods. In the first experiment, samples were stored in the dark at 4ºC or in a freezer at –12ºC. In the second, samples were stored at room temperature, at 4ºC, at –18ºC, or with the addition of chloroform or thymol.

### Collector Design

Four different rain collectors were set up at CEH Edinburgh (15 km southwest of Edinburgh). All were mounted 1.5 m above ground, in a square of side 5 m, in the middle of a grass field. Funnels and collecting bottles were constructed from polypropylene (P), from borosilicate glass (G), and two with stainless steel funnels and glass bottles (SG1, SG2). Thymol (100 mg) was added to all but the SG2 sampling bottles. Samples were collected approximately weekly, depending on rain amount, and filtered before shipping to the laboratory in Cumbria for analysis. No attempt was made to quantify any particulate organic N removed from the samples by filtration. The nonpreserved sample (SG2) was split, and thymol was added to one of the split samples to test for the need for a biocide during transport and storage prior to analysis. Samples were analysed for $NH_4^+$ (by both ion chromatography and colorimetry), $NO_3^-$ (ion chromatography), and total N (persulphate digestion/colorimetry).

### Sampling Network Across the U.K.

Aluminium frames were erected to house stainless steel funnels draining to 1 or 2 l glass collection bottles. Sample bottles were placed in the collectors with the addition of 100 mg thymol, and left for 1 week before sampling. Samples were filtered under vacuum immediately, through preashed glass fibre filters, before shipping to the laboratory in low-density polyethylene bottles. The locations of the sampling sites are given in Table 1.

## RESULTS

### Chemical Analyses

#### *Inorganic Components*

Both $NO_3^-$ methods were found to be accurate, robust, of adequate sensitivity, and free from interferences from other N-containing compounds and chemical preservatives. Similarly, quantification of $NH_4^+$ by ion chromatography was accurate and free from interferences from other N-containing compounds. As a standard clean-up protocol for both anion and cation ion chromatography procedures, $C_{18}$ stationary phase cartridges are used to reduce the organic loading (e.g., from chemical treatments or

**TABLE 1**
**Long-Term (>1 Year) U.K. Network Measurement Sites**

| Site | Location | Altitude (m) | Annual Rainfall (mm) |
|---|---|---|---|
| Cairngorms | NE Scotland | 325 | 1100 |
| Bush | SE Scotland | 200 | 800 |
| Merlewood | NW England | 90 | 1200 |
| Moorhouse | N England | 560 | 1900 |
| Climoor | N Wales | 500 | 1990 |
| Norwich | SE England | 130 | 650 |
| Dorset | SW England | 50 | 1000 |

dissolved organic compounds) onto the columns, to ensure the long-term integrity of the system. Although direct tests showed that ammonium was not absorbed from aqueous solutions, we found some evidence to suggest that a portion may be retained on the stationary phase when chemical preservatives such as thymol are present in solution (see below).

The indophenol blue colorimetric method shows detectable positive responses from other N-containing compounds. Free amino acids reacted to form an indophenol blue complex. For glycine, a 36% response was measured at a concentration of 10 µM, for methionine a 55% response was observed at the same concentration, whilst urea and 5-iminodiacetic acid did not react to form a coloured complex. We have confirmed by ion chromatography that this response is not due to traces of ammonium ions in the test compounds. These findings confirm those of an earlier study[9] that found 47% response to glycine from a similar indophenol blue continuous flow method.

### *Total Dissolved Nitrogen*

The UV oxidation method was effective (recoveries >90%) for model compounds[8], but could not be used for chemically preserved rain samples because the presence of the thymol preservative drastically reduced the potential to oxidise the organic N compounds and produced cloudy solutions.

Persulphate oxidation was capable of quantifying N in the presence of chemical preservatives, providing the concentrations of thymol were controlled within a narrow range (see below). Persulphate oxidised all or a portion of the study compounds. Good recoveries were achieved from the inorganic compounds (103%), but were lower for amino acids (80%) and for urea (68%) for concentrations in the range 5 to 10 µM.

A wide range of N-containing compounds dissolved in water was analysed by the ANTEK high-temperature chemiluminescence instrument, to check for equimolar response and linearity. There was a significant effect of the carrier solvent used, presumably because of changes in the oxidation furnace, but the equipment was not optimised for each carrier. The response of $NH_4^+$, $NO_3^-$, urea, methionine, and glycine was the same to within the precision of the solutions, but greatly reduced sensitivity was observed for complex heterocyclic compounds such as 1,10-phenanthroline. The response was linear up to at least 150 µM. A filtered rain sample can be analysed without further preparation in about 3 min, limited only by the autosampler frequency. The detection limit (3 × noise) was ca. 1 ng N for the conditions used, equivalent to a concentration of 3 µM; a larger sample volume would lower the detection limit.

A comparison of analytical methods using rain samples (Fig. 1) illustrates the conclusions from the above. $NH_4^+$ analysis by the indophenol blue method overestimated the concentration as measured by ion chromatography. The ANTEK method gave slightly higher readings for total N than persulphate oxidation, consistent with a less than total recovery of organic N by the latter. The combination of persulphate oxidation for total N and the indophenol blue method for $NH_4^+$ therefore biases the estimate of organic N significantly. If the ANTEK method and ion chromatography are assumed to be accurate, the DON concentrations in the samples averaged 16 µM, but the average difference between persulphate digest and DIN using the indophenol blue method for $NH_4^+$ was only 5 µM.

### Choice of Biocide

#### *Determination of Optimum Biocide Concentration*

The results are shown in Fig. 2. Although DON concentrations appeared to be relatively stable over 1 week, concentrations of $NH_4^+$ were smaller in samples with no biocide added, or with biocide concentrations less than 100 mg $l^{-1}$. Above 200 mg $l^{-1}$, thymol interfered with the persulphate digestion for total N, and at the highest concentration used (1 g $l^{-1}$) the digest was strongly discoloured (not shown in Fig. 2).

### *Storage Experiments*

#### *Long-Term (9-Month) Storage*

Results from the analysis of the sample at intervals over 9 months are shown in Fig. 3. The first four bars/clusters on each chart are data for aliquots stored in the dark at 4°C, and the fifth set of bars show the final results for frozen aliquots. $NO_3^-$ and $NH_4^+$ (DIN) concentrations decrease in the refrigerated samples (9%), as well as in the frozen samples (6%, not significantly different from 4°C). Total N determinations in the refrigerated samples were extremely variable between analyses, although this reflects the comparatively low reproducibility within analytical batches

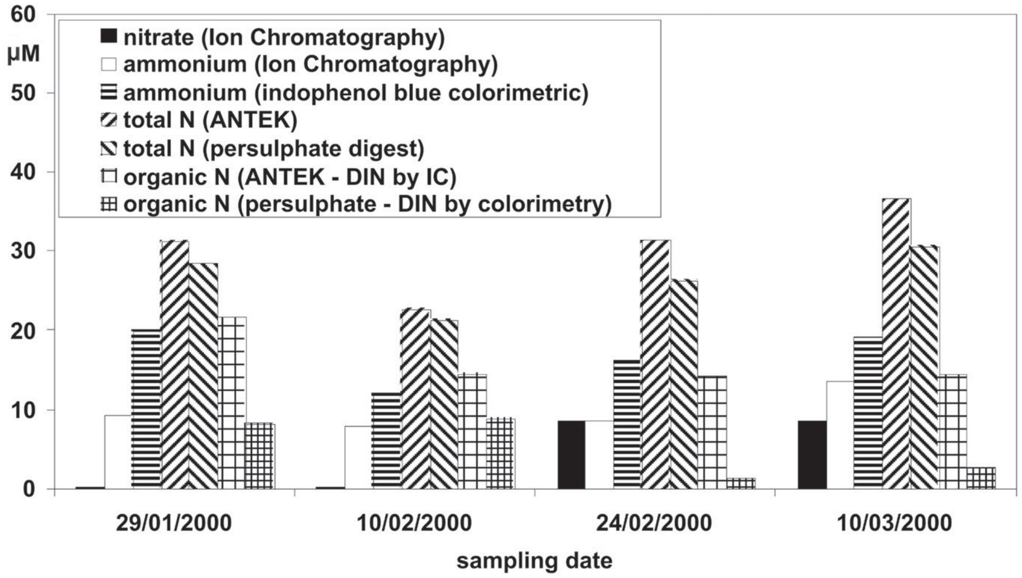

**FIGURE 1.** Comparison of analytical methods (Bush Estate, January-March 2000). Samples without thymol.

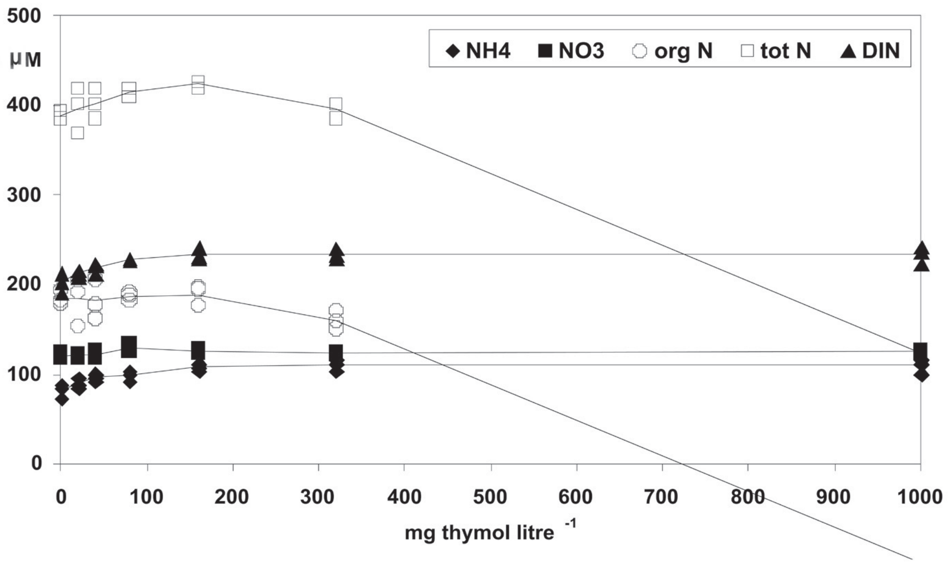

**FIGURE 2.** Test of thymol as biocide. Rain sample analysed after storage for 1 week with different amounts of thymol added.

(±19% for triplicate total N analysis of refrigerated samples), leading to great uncertainty in the calculated organic nitrogen (ON) determinations. Analytical precision problems in samples, where ON is a small fraction of total N, have been discussed previously[8]. The total N determination in the frozen samples was more consistent, with typical within-batch variability of <2% for DIN and ~5% for total N. Even in these conditions, however, the propagated uncertainty in the calculation of DON by difference can be very large when the fraction of DON is small.

### *Short-Term (3-Month) Storage*

In the first experiment, a range of samples analysed over a shorter storage period (at 4°C) showed no systematic trends in concentration for inorganic or organic N. Samples were analysed immediately, after 1 month and after 2 months.

In the second experiment, there was no change in $NO_3^-$ concentrations in any of the samples on storage for 22 days, even at room temperature. In contrast, samples stored at room tempera-

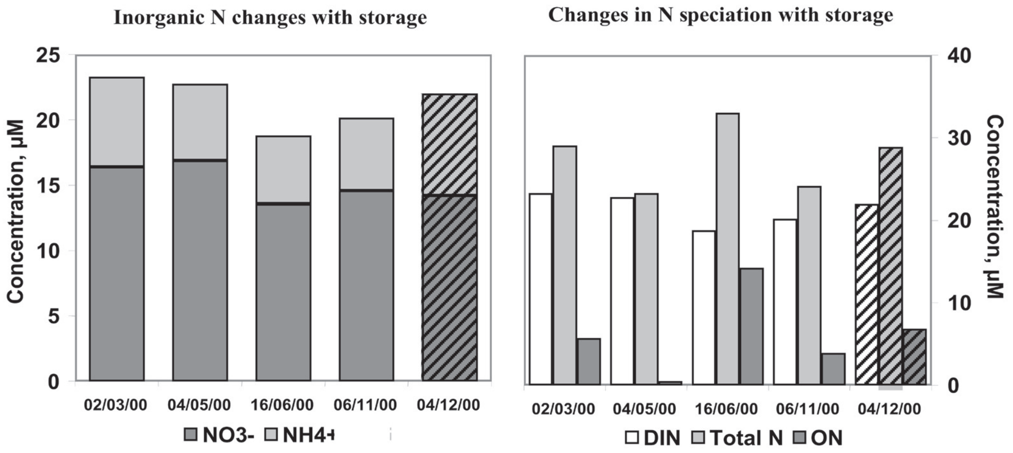

**FIGURE 3.** Effect of strage of 9 months at 4ºC on N content of a rain sample.

ture lost 20% $NH_4^+$ (measured colorimetrically), with statistically significant differences ($p = 0.002$; ANOVA with five treatments, two replicates) appearing after 10 days. There were no significant changes in $NH_4^+$ concentrations in the other samples. The analyses for $NH_4^+$ using solid phase extraction (to remove organics) followed by ion chromatography gave variable results, indicating that the clean-up procedure may lead to random loss of $NH_4^+$ in the presence of organic biocides. The concentration of organic N compounds in the study sample was low (approximately 8 µM). Therefore it was not possible to determine any statistically significant change in concentration with time, but generally the trends indicated no decline on storage, regardless of preservation treatment.

### Effects of Different Collector Materials

Statistical analysis showed no significant effect of bottle or funnel material on any of the measured concentrations, and no effect of the biocide, after taking account of the large variation in concentrations between sampling dates. Under the conditions at this site, the use of a biocide, while advisable, may not be strictly necessary. The finding that there is no systematic pattern associated with the use of different collector materials contrasts with that of Scudlark et al.[5], who found a systematic undersampling of total organic N in precipitation using plastic, relative to steel, collectors. This may be because these authors measured total N on unfiltered samples, which may have contained suspended organic N particles that were retained by the plastic containers.

The average concentrations of the different N components of rain in the five samples each week from Bush (near Edinburgh) are shown in Fig. 4, and demonstrate the week-to-week variations in concentration, and in the calculated amounts of organic N.

### U.K. Network for Measuring DON in Precipitation

The volume-weighted mean concentrations measured in precipitation collected between June and November 2000 in this study are shown in Table 2. Total N concentrations were smallest at the remote sites of Cairngorm and North Wales (Climoor), and greatest at East Stoke (Dorset) and Norwich. Concentrations of oxidised and reduced N were in similar proportion at all but one of the network sites. In contrast to the other sites, the oxidised form of N dominated precipitation collected at Norwich, perhaps through local contamination by nitric acid vapour from the university laboratories.

Organic N concentrations were greatest at Moorhouse, Merlewood, and East Stoke, and small at the other sites, especially Norwich, Cairngorm, and Climoor. Mean site values range from 0 to 37 µM , corresponding to a national mean of 21% organic N in solution.

Samples collected at Moorhouse show differences in the organic N fraction between the samples with and without thymol[10] as a biocide. It appears that the addition of thymol has prevented the loss of $NH_4^+$ and organic N from the samples, but there was a pattern of systematic contamination of the thymol-containing sampler by bird faeces (obviously contaminated samples were not included in the data in Table 2).

For the network samples, analysis of $NH_4^+$ in precipitation by both ion chromatography and colorimetric techniques showed generally good agreement. The benefit of the biocide thymol, in preventing losses of $NH_4^+$, is offset by the greater variability in the persulphate method for analysis of total N, and the interference in the UV method.

The network measurements represent a significant increase in the available organic N data for the U.K. A review paper is in preparation that includes an assessment of regional trends in the contribution of organic N to rainwater N (based mainly on reports from Europe, U.K., and North America), and a summary of what is known about the rainwater concentrations of the main compound groups that comprise bulk organic N. On average, over half of the organic N in coastal and continental rain is in the form of amides (amino acids and polypeptides), consistent with the reported concentrations of Kjeldahl N. Urea is also a significant component (~5 to 10%) of rainwater organic N, although there is a great deal of spatial and probably also seasonal variation[11]. Other reduced N compounds (primarily the amines) are minor constituents (~5%). These compound classes are predomi-

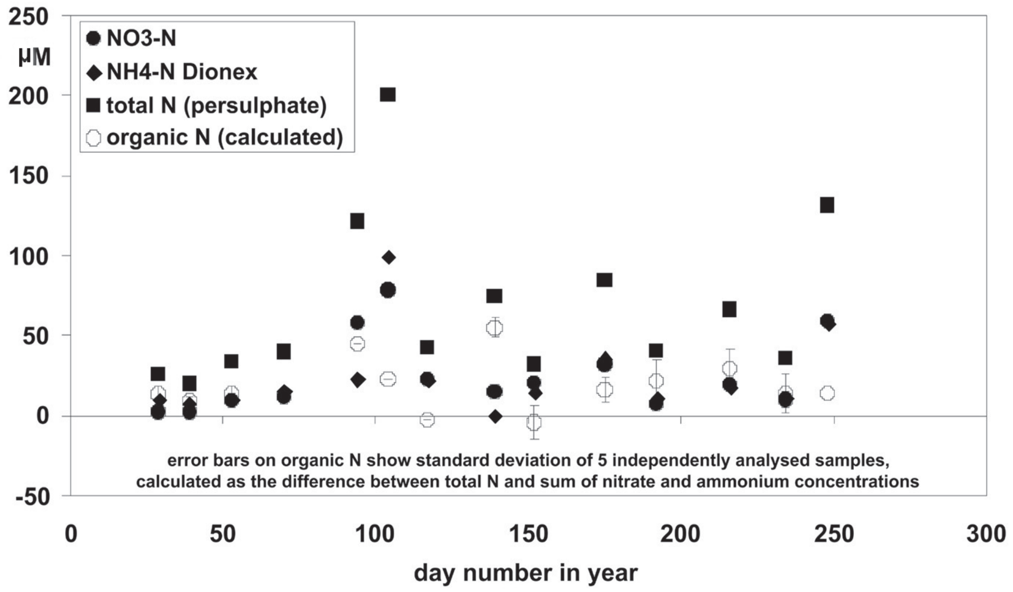

**FIGURE 4**. Concentrations of dissolved N in rain, Bush Estate, 2000.

**TABLE 2**
**U.K. Network N Concentrations in Precipitation, June–November 2000**

| μM | $NO_3^-$ | $NH_4^+$ Colorimetric | Total N Persulphate | Organic N | Organic N (%) | No. of Samples |
|---|---|---|---|---|---|---|
| Cairngorms | 18 | 9 | 27 | 0 | 0 | 5 |
| Bush | 20 | 24 | 55 | 11 | 26 | 7 |
| Merlewood | 20 | 34 | 84 | 31 | 21 | 10 |
| Moorhouse | 20 | 31 | 81 | 31 | 32 | 8 |
| Moorhouse (ECN)[a] | 17 | 15 | 38 | 6 | 17 | 8 |
| North Wales | 9 | 8 | 25 | 8 | 31 | 4 |
| Norwich | 84 | 53 | 121 | 0 | 0 | 5 |
| Dorset | 35 | 39 | 112 | 37 | 37 | 8 |

[a] The ECN Moorhouse precipitation collection vessel did not contain a biocide.

nantly biogenic/agricultural in origin. The largely anthropogenic substituted polycyclic and heterocyclic aromatic compounds and the peroxyacetyl nitrate (PAN) derivatives and other organic nitrates are also minor constituents (~1%). Published reports usually have concentration data only for specific individual compounds, rather than for the full range of homologues, so the overall contribution from these classes is still uncertain.

Although the above discussion considers only wet-deposited N, there is likely also to be a contribution of organic N, both as particles and gases, to dry deposition. We know of no direct measurements of dry deposition of organic N, but analyses of airborne particulates suggest that the fraction of organic N can be large[13,15]. However, if the organic N is predominantly associated with submicron aerosol, as we have found in some of our studies in Hawaii[16], then the contribution to dry deposition may still be small, given the slow deposition rates of this size of aerosol.

### SUMMARY AND CONCLUSIONS

- Both colorimetric and ion chromatographic methods for analysis of $NO_3^-$ in rainwater are robust in the presence of thymol as a preservative, but $NO_3^-$ concentrations appear to be stable over several months, even in the absence of a chemical preservative.
- $NH_4^+$ concentrations may be overestimated by the indophenol blue method, resulting in systematically low organic N values calculated by difference. The clean-up of rain samples prior to ion chromatography may lead to random retention of $NH_4^+$ in the presence of thymol. However, significant losses of $NH_4^+$ were observed during storage of rain samples without an added biocide or freezing, showing the need for a chemical preservative during sampling.

- The ANTEK chemiluminescence analyser offers rapid and accurate determinations of total dissolved N in water samples, with detection limits of ca. 1 ng N per injection.
- The addition of thymol as biocide had no detectable effect on overall composition at one site, but a large effect at another. Routine use of a biocide is advised. The optimum thymol concentration is 100 mg $l^{-1}$.
- No difference was found in the *dissolved* organic N content of rain sampled in collectors made of glass, steel, or polypropylene.
- DON contributes about 20% of total N in rain over the U.K., but with large geographical and temporal variability.

### ACKNOWLEDGEMENTS

This research was funded by the U.K. Natural Environment Research Council's GANE Programme.

| | | |
|---|---|---|
| Received: | July | 11, 2001 |
| Revised: | September | 17, 2001 |
| Accepted: | October | 5, 2001 |
| Published: | November | 7, 2001 |

### BIOSKETCH

**J. Neil Cape** is Deputy Section Leader of the Trace Gas/Air Pollution Section at the Centre for Ecology and Hydrology, Edinburgh, Scotland. He received his B.Sc. (Hons) in Chemistry at St. Andrews, his Ph.D. in Physical Chemistry at Cambridge, and his D.Sc. at St. Andrews. He is a Fellow of Royal Society of Chemistry, a member of the British Ecological Society, and a Chartered Chemist. Dr. Cape's research interests include the interactions of trace gases and particles, both organic and inorganic, at the earth's surface, and the processes that control the exchange of material between vegetation and the atmosphere. Included are the responses of plants to pollution, and the interaction of plant physiology and biochemistry with the chemical processes occurring on leaf surfaces.
